# Factors influencing the presence of sand flies in Majorca (Balearic Islands, Spain) with special reference to *Phlebotomus pernicious*, vector of *Leishmania infantum*

**DOI:** 10.1186/1756-3305-7-421

**Published:** 2014-09-04

**Authors:** M Magdalena Alcover, Cristina Ballart, Joaquina Martín-Sánchez, Teresa Serra, Soledad Castillejo, Montserrat Portús, Montserrat Gállego

**Affiliations:** Laboratori de Parasitologia, Facultat de Farmàcia, Universitat de Barcelona (Spain), Barcelona, Spain; Centre de Recerca en Salut Internacional de Barcelona (CRESIB), UB-Fundació Clínic, Barcelona, Spain; Departamento de Parasitología, Facultad de Farmacia, Universidad de Granada, Granada, Spain; Grup d’Estudi de les Malalties Emergents, Institut Universitari d’Investigació en Ciències de la Salut, IUNICS, Mallorca, Spain

**Keywords:** Leishmaniosis, *Phlebotomus perniciosus*, Risk factors, Majorca Island

## Abstract

**Background:**

Although the Mediterranean island of Majorca is an endemic area of leishmaniosis, there is a lack of up-to-date data on its sand fly fauna, the last report dating from 1989. The aim of the present study was to provide information on the current sand fly distribution, the potential environmental factors favoring the presence of *Phlebotomus perniciosus* and which areas are at risk of leishmaniosis.

**Methods:**

In July 2008 sand fly captures were carried out in Majorca with sticky castor oil interception traps. The capture stations were distributed in 77 grids (5x5 km^2^) covering the entire island. A total of 1,882 sticky traps were set among 111 stations. The characteristics of the stations were recorded and maps were designed using ArcGIS 9.2 software. The statistical analysis was carried out using a bivariate and multivariate logistic regression model.

**Results:**

The sand fly fauna of Majorca is composed of 4 species: *Phlebotomus perniciosus*, *P sergenti*, *P. papatasi* and *Sergentomyia minuta. P. perniciosus*, responsible for *Leishmania infantum* transmission*,* was captured throughout the island (frequency 69.4 %), from 6 to 772 m above sea level. Through logistic regression we estimated the probability of *P. perniciosus* presence at each sampling site as a function of environmental and meteorological factors. Although in the initial univariate analyses the probability of *P. perniciosus* presence appeared to be associated with a wide variety of factors, in the multivariate logistic regression model only altitude, settlement, aspect, drainage hole construction, adjacent flora and the proximity of a sheep farm were retained as positive predictors of the distribution of this species.

**Conclusions:**

*P. perniciosus* was present throughout the island, and thereby the risk of leishmaniosis transmission. The probability of finding *P. perniciosus* was higher at altitudes ranging from 51 to 150 m.a.s.l., with adjacent garrigue shrub vegetation, at the edge of or between settlements, and in proximity to a sheep farm.

## Background

The Balearic Islands in the Mediterranean region are considered endemic for both human and canine leishmaniosis, although the presence and prevalence of the diseases varies among the islands [1]. The first data on human leishmaniosis in the Balearic Islands date from 1925 [2], while canine leishmaniosis was first reported in 1989 [3], in both cases in the island of Majorca, where most studies have been conducted.

In certain regions of Spain, human leishmaniosis is an endemic and notifiable disease, including in the Balearic Islands, which in some years have seen the highest registered incidence in Spain (4.72 and 4.59/100,000 in 2005 and 2006 respectively) [4]. Between 7 and 33 cases are declared in Majorca every year [5, 6]. As in other parts of Spain, the disease is under-reported, especially cases of cutaneous leishmaniosis [7]; cases of human cryptic leishmaniosis have also been described [8]. Little information is available on the origin of cases [6, 8].

The heterogeneous distribution and prevalence of canine leishmaniosis (CanL) ranges from 0% to 45% among different studies and islands [9–11]. A study conducted by the sanitary authorities in Majorca gave a prevalence of 14.4 % [3]. Veterinarians answering a questionnaire on CanL trends in Majorca thought the disease was stable [1] and that autochthonous cases continue to occur, as has been previously described [3, 11].

Data on sand fly distribution in the Balearic Islands is scarce [10, 12–15]. The most recent data for Majorca corresponds to studies performed in 1987 and 1989 [14], but do not include information about the distribution and density of the different sand fly species throughout the island.

The aim of the present study was to obtain up-to-date entomological data by standardized methods that could be compared with data reported by other teams in different geographical areas of Europe and used in future entomological studies, including those on climate change. In addition, the extensive capture of the vector in the island could provide information on the environmental factors that may potentially favour the presence of *P. perniciosus* and also which areas are at risk of leishmaniosis.

## Methods

### Area of study

The study was carried out on the island of Majorca (Spain), located at 39°15’ to 39°60’N, 2°20’ to 3°30’E. Majorca is the largest of the Balearic Islands, covering 3,640 km^2^ and with a coastline of 623 km. Altitudes range from sea level to 1,445 m.a.s.l., most of the island (78.8%) being below 200 m.a.s.l. and only 6.3% above 500 m.a.s.l. The highest mountainous area is the Serra de Tramuntana in the north, which runs parallel to the west coast, protecting the island from the prevailing west and northwest winds. Bordering the low central plain in the southeast is the Serra de Llevant, with a maximum altitude of 509 m.a.s.l. [16].

The climate is typically Mediterranean, with long periods of invariability. The mean annual temperature is about 16–17°C, except in the Serra de Tramuntana, where it drops to 13°C. In the coldest period (1–3 months), the average temperature is about 5-10°C, with an average minimum on winter nights of 5–9°C, while in the hottest period (5–8 months) it is above 15-20°C, with an average diurnal maximum of 29-31°C. The mean relative humidity is 74%. Annual rainfall oscillates from a maximum in autumn (66.9 mm) to a minimum in summer (8.6 mm), with an annual average of 481.6 mm. Considerable differences exist between mountainous regions (up to 1,200 mm) and the arid south (less than 400 mm).

Holm oak (*Cyclamini-Quercetum ilicis*) grows everywhere on the island below 1000 m.a.s.l, but under the influence of man it has largely been replaced by pine (*Pinus halepensis*), which is now the dominant woodland tree, including all well-conserved beaches. In areas below 500–700 m.a.s.l., with annual precipitations of less than 600 mm, the wild olive tree predominates, while above 1000 m.a.s.l, the vegetation is low and adapted to strong winds. The extensive cultivated land consists principally of almond and olive trees, vineyards and cereals.

The island has two bioclimatic zones: meso-Mediterranean (T: average annual temperature 13–17°C; m: average minimum temperature of the coldest month -1 to -4°C; M: average maximum temperature of the coldest month 9 - 14°C; Ti: thermicity index 210–350), where oaks predominate (*Cyclamini-Quercetum ilicis*), and thermo-Mediterranean (T: 17–19°C; m: 4–10°C; M: 14 - 18°C; Ti: thermicity index from 350–470) with maquis (*Cneoro-Ceratonietum*)) [16, 17].

### Capture of sand flies

**Figure 1:**
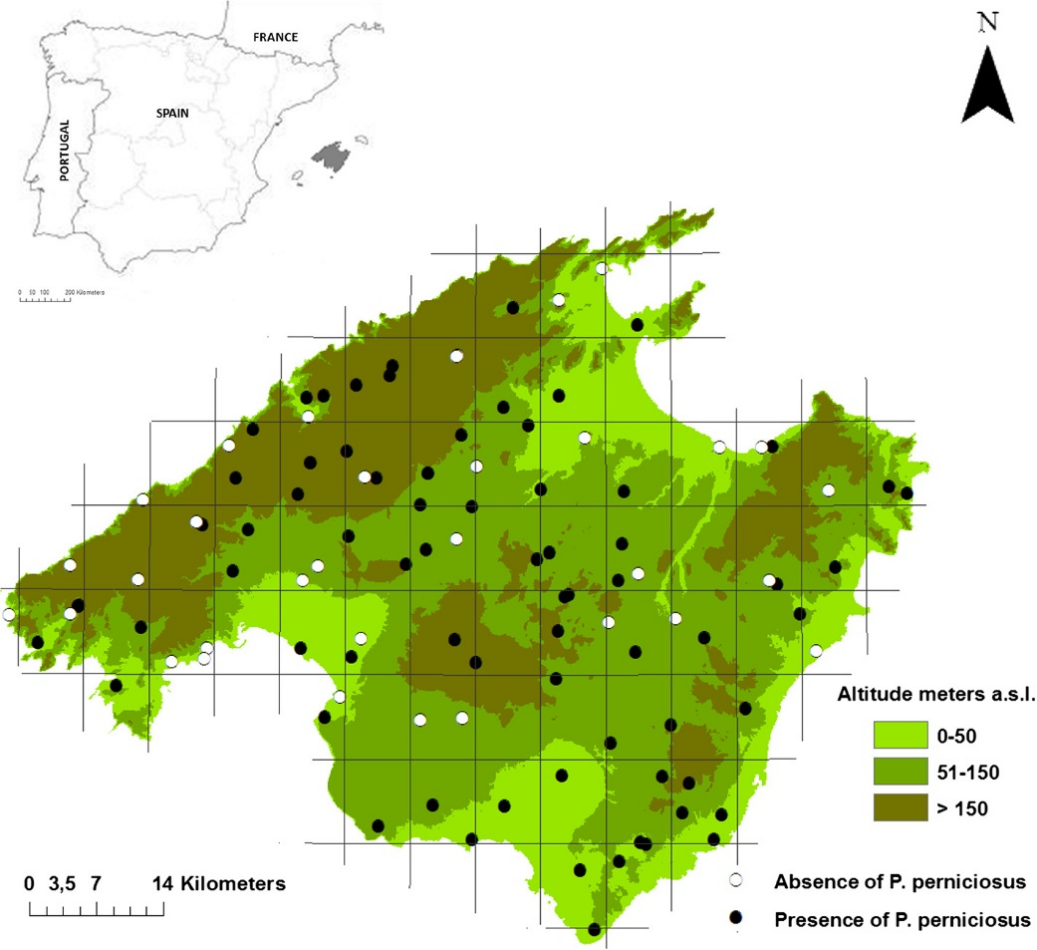
**Sampling sites in Majorca showing the presence/absence of** ***P. perniciosus*** **and altitudinal ranges of the island.**

In July 2008 sand fly captures were carried out in Majorca with sticky castor oil interception traps (20×20 cm) set for 4 days according to the standardized methodology implemented in the EDEN project (EU) [18–21]. The sampling sites consisted of holes used to drain embankments or containment walls, which were considered to be likely diurnal resting places for adult sand flies [22]. The capture stations were distributed in 77 grids (5×5 km^2^), almost one station per grid, covering the entire island. A total of 1,882 sticky traps were set, representing an adhesion surface of 150.56 m^2^ distributed among 111 stations (Figure 1).

### Data collection and environmental and meteorological variables

The characteristics of the stations, including location, habitat, environment and fauna, were recorded on a PDA (Palm Tungsten T5) using Pendragon Form v.5.0 software (PSC, Libertyville, IL, USA) and GPS (Tom Tom Wireless GPS MK II). Maps were designed using ArcGIS 9.2 software (ESRI, Redlands, CA, USA).

Climate variables were provided by the Spanish Meteorological Agency (AEMet) from the 43 meteorological stations in the study area. Different periods were considered for the meteorological variables: i) Period 1 (Sampling Day 1, when traps were set, to Day 4, when traps were recovered) and ii) Period 2 (the month before Sampling Day 1). Climate data from the nearest meteorological station were assigned to each sampling site for periods 1 and 2 using the spatial join-and-relate tool of ArcGis v.9.2 software and included: wind speed (Km/h), mean relative humidity (%), average rainfall (mm), and mean daily T (°C). The average minimum T (°C) in winter was also assigned.

Altitude data for each geocoded collection site were extracted from a 90 m resolution CGIAR Digital Elevation Model [23] using ArcGIS 9.2 software.

The presence of animals was studied in two ways: taking into account the animals or animal signs observed during captures, and using databases provided by the Col · legi Oficial de Veterinaris de les Illes Balears (canine census and livestock farms). In the latter, data from the closest station were assigned for each sampling site as described for the meteorological stations. A human census was obtained from municipal data.

### Sand fly processing and identification

Sand flies were processed as previously described [1]. Briefly, sand flies were removed from the sticky traps with a brush and fixed in 96% ethanol and then in 70% alcohol until identification. Males and *Sergentomyia* spp. females were observed and identified under the stereo microscope. Females of the genus *Phlebotomus* were mounted on glass slides in Hoyer medium and identified on the basis of morphological characteristics in an optical microscope using the keys of Gállego [24].

### Statistical analysis

For the study 57 variables were taken into account, including the habitat and environmental characteristics of the capture stations, fauna, demography and climate.

Bivariate logistic regression studies were conducted using the SPSS 20.0 software for Windows, with all the independent variables set against the presence/absence of *P. perniciosus* as the dependent variable. The majority were used as categorical variables, except those related to meteorological conditions. Continuous variables such as the human and canine census were categorized in the search for association with the dependent variable. The possibility of interaction and/or confusion between different variables was examined by constructing and comparing different logistic regression models [19].

To construct the multivariate model, all the variables with p <0.2 in the bivariate study were used. In the final multivariate model, variables with p ≤0.05 were retained.

## Results

88.2 % of the traps placed on the island of Majorca were recovered, representing a surface of 135.68 m^2^. A total of 14,412 specimens were captured, with 4 species identified (Table 1): *Phlebotomus pernicious*, *P. sergenti*, *P. papatasi* and *Sergentomyia minuta*.

**Table 1:** **Quantitative results of the sand fly fauna of Majorca. M: males, F: females**

Species	Sex. ratio (M:F)	Abundance (%)	Density (n/m ^2^)	Frequency (%)
*P. perniciosus*	4:1	6.3	6.72	69.37
*P. sergenti*	24:1	0.2	0.18	12.61
*P. papatasi*	3:0	0.2 x 10^-3^	0.02	0.9
*S. minuta*	1.4:1	93.5	99.3	92.8

Among the mamophilic species, *P. perniciosus* was captured throughout the island in 77 of the 111 stations prospected, at 6 to 772 m.a.s.l. (Figure 1), with climate conditions during the capture period of 19.6-27.4°C, 55.5-86.4% relative humidity, 0–42 mm pluviometry and 3.1-17.1 km/h wind speed. *P. sergenti* and *P. papatasi* were captured in only 14 and 1 of the stations, respectively, and always in a low number. *P. ariasi* was not found anywhere on the island.

### Bivariate analysis

The bivariate analysis of the factors associated with the presence of *P. perniciosus* gave results of p <0.2 for 24 of the variables, which were taken into account in the multivariate analysis. 12 of these variables showed significant association (p <0.05) with the sand fly presence in both bivariate and multivariate analyses (Table 2).

**Table 2:** **Risk factors for the presence of** ***Phlebotomus perniciosus*** **in Majorca: Bivariate logistic regression model**

	Number of stations (111)	Odds ratio (I.C. 95 %)	p - Value
**Altitude (m.a.s.l.)**			**0.063**
0-50	28	**Ref.**	
51-150	60	3.133 (1.195 – 8.214)	0.020
>150	23	1.625 (0.522 – 5.055)	0.402
**Settlement**			
Within settlement	21	**Ref.**	
Edge of/between settlements	90	5.339 (1.950 – 14.617)	0.001
**Type of roadway**			**0.228**
Paved public road	46	**Ref.**	
Paved drive	41	2.903 (1.100 – 7.658)	0.031
Unpaved track	9	2.463 (0.460 – 13.182)	0.292
Garden (in rural village and other settlement)	5	2.815 (0.291 – 27.206)	0.371
Property (farm and other)	10	1.056 (0.262 – 4.258)	0.939
**Site category**			**0.421**
Embankment drainage holes	19	**Ref.**	
Wall drainage holes (not embankment)	26	2.111 (0.204 – 0.843)	0.031
Other holes in walls (not embankment)	47	0.308 (0.062 – 1.522)	0.148
Natural rock crevices	3	0.235 (0.014 – 3.917)	0.313
Farm buildings (holes)	13	0.264 (0.040 – 1.735)	0.166
Sewer/drainage openings	3	-	0.999
**Aspect**			**0.26**
Other (all orientations except south-east and west facing)	73	**Ref.**	
South-east facing	15	2.990 (0.623 – 14.350)	0.171
West facing	23	0.716 (0.271 – 1.892)	0.500
**Slope**			**0.843**
None	79	**Ref.**	
Shallow (<10 %)	30	1.018 (0.407 – 2.546)	0.969
Steep (>10 %)	2	0.436 (0.026 – 7.270)	0.563
**Shelter**			**0.776**
Not sheltered	93	**Ref.**	
Sheltered	17	1.548 (0.465 – 5.149)	0.476
Unsure	1	-	1,000
**Water course**			
None	105	**Ref.**	
Natural	6	0.419 (0.080 – 2.191)	0.303
**Wall construction**			**0.013**
Stone without mortar	48	**Ref.**	
Stone/mortar	16	0.338 (0.101 – 1.133)	0.079
Brick/mortar	30	0.263 (0.097 – 0.714)	0.009
Other	17	1.974 (0.386 – 10.089)	0,414
**Drain hole construction**			
Plastic pipe	35	**Ref.**	
Other (unlined, cement pipe)	76	2.250 (0.964 – 5.249	0.061
**Hole interior**			**0.961**
Bare	33	**Ref.**	
Dusty (bare)	68	0.784 (0.313 – 1.966)	0.604
Dusty (with vegetation)	3	0.750 (0.060 – 9.319)	0.823
Soil (with vegetation)	7	0.938 (0.153 – 5.728)	0.944
**Vegetation on the wall**			
No	86	**Ref.**	
Yes	25	1.529 (0.550 – 4.251	0.416
**General environment**			**0.02**
Rural village	48	**Ref.**	
Rural agriculture and forestry	45	2.977 (1.095 – 8.091)	0.032
Coastal village	8	0.548 (0.122 – 2.475)	0.435
Other settlement (non rural or non coastal village)	10	0.366 (0.090 – 1.478)	0.158
**General vegetation (100 m – 1Km)**			**0.178**
Aleppo pine	51	**Ref.**	
Evergreen oaks	3	0.273 (0.023 – 3.219)	0.302
Garrigue shrubs	38	2.416 (0.888 – 6.575)	0.084
None	19	0.935 (0.313 – 2.795)	0.904
**Adjacent flora**			**0.02**
Aleppo pine and evergreen oaks	30	**Ref.**	
Garrigue shrubs	40	14.529 (2.949 – 71.587)	0.001
None	41	0.885 (0.343 – 2.284)	0.801
**Land cover (Corine)**			**0.006**
Urban area	33	**Ref.**	
Agricultural area	62	5.525 (2.113 – 14.448)	<0.001
Forest area	15	1.594 (0.462 – 5.497)	0.461
Humid area	1	-	1.000
**Arable**			**0.107**
Cereals	35	**Ref.**	
Root crop	2	0.167 (0.009 – 3.118)	0.231
Other (non cereal or root crop)	6	0.333 (0.048 – 2.328)	0.268
None	68	0.269 (0.093 – 0.781)	0.016
**Garden**			**0.385**
Grass, shrubs and trees	36	**Ref.**	
Paved hard surface	8	1.333 (0.276 – 6.442)	0.720
Orchard	56	2.187 (0.903 – 5.294)	0.083
None	11	-	0.999
**Bioclimatic**			
Meso-Mediterranean	63	**Ref.**	
Thermo-Mediterranean	48	1.130 (0.499 – 2.559)	0.770
**Demographic data**			
**Humans**			
≥ 688,5	106	**Ref.**	
≤ 688,4	5	0.276 (0.044-1.731)	0.169
**Canine**			
≤ 1989	56	**Ref.**	
≥ 1990	55	0.693 (0.308-1.561)	0.376
Animals seen**			
**Dogs**			
Yes	56	0.583 (0.258 – 1.321)	0.196
**Cats**			
Yes	9	1.600 (0.315 – 8.135)	0.571
**Pet animals (dogs and cats)**			
Yes	56	0.615 (0.272 – 1.391)	0.243
**Equine**			
Yes	7	2.789 (0.323 – 24.107)	0.351
**Cattle**			
Yes	2	0.434 (0.026 – 7.153)	0.560
**Goat**			
Yes	3	0.880 (0.077 – 10.047)	0.918
**Sheep**			
Yes	23	1.769 (0.597 – 5.242)	0.303
**Pig**			
Yes	1	-	1.000
**Farm animals seen**			
Yes	32	1.472 (0.582 – 3.721)	0.414
**Rabbit**			
Yes	2	-	0.999
**Chicken**			
Yes	13	2.667 (0.558 – 12.750)	0.219
**Duck**			
Yes	2	-	0.999
**Pigeon**			
Yes	5	0.649 (0.103 – 4.070)	0.644
**Pen animals seen** (Chicken, duck and pigeon)			
Yes	17	1.523 (0.458 – 3.066)	0.492
Livestock farms near***			
**Horse**			
Yes	44	0.540 (0.238 – 1.225)	0.140
**Sheep**			
Yes	58	2.720 (1.177 – 6.289)	0.019
**Goat**			
Yes	9	1.600 (0.315 – 8.135)	0.571
**Pigs**			
Yes	34	1.087 (0.450 – 2.624)	0.853
**Rabbit**			
Yes	7	0.304 (0.064 – 1.441)	0.134
**Bovine**			
Yes	4	0.427 (0.058 – 3.163)	0.405
**Chicken**			
Yes	23	0.786 (0.297 – 2.080)	0.628
**Turkey**			
Yes	4	0.136 (0.014 – 1.358)	0.089
**Pigeon**			
Yes	7	0.155 (0.028 – 0.842)	0.031
**Pheasant**			
Yes	1	-	1
**Quail**			
Yes	1	-	1
**Partridge**			
Yes	1	-	1
**Bees**			
Yes	5	0.099 (0.011 – 0.919)	0.042
Meteorological variables (continuous)*			
Wind period 1	3.1 – 17.1	0.937 (0.856 – 1.025)	0.157
Wind period 2	3.1 – 15	0.952 (0.854 – 1.062)	0.381
Humidity period 1	55.5 – 86.4	0.956 (0.907 – 1.008)	0.099
Humidity period 2	74.7 – 96.7	0.952 (0.881 – 1.028)	0.207
Rainfall period 1	0 – 42	0.954 (0.897 – 1.013)	0.126
Rainfall period 2	0 – 511	1.000 (0.996 -1.004)	0.936
Temperature period 1	19.6 – 27.5	0.911 (0.722 – 1.149)	0.432
Temperature period 2	19.8 – 26.2	0.974 (0.756 – 1.253)	0.835
Wintry temperature	-2.6 – 5.3	1.068 (0.857 – 1.331)	0.560

Dependent variable presence/absence of *P. perniciosus*. Ref. Reference category. C. I. = Confidence interval. Period 1: sampling day 1(traps set) today 4 (traps recovered). Period 2: the month before sampling day 1. *N is substituted by minimum and maximum values. **Reference category Animals seen: No. ***Reference category Livestock farms near: No.

The probability of capturing *P. perniciosus* was significantly higher at 51 – 150 m.a.s.l. (O.R. = 3.13), at the edge of or between settlements (O.R = 5.3), on a paved drive (O.R. = 2.90), in a wall drainage hole (not embankment) (O.R. = 2.11), in a general rural agricultural or forestry habitat (O.R. = 2.98), with adjacent flora of garrigue shrubs (O.R. = 14.53), in an agricultural area (O.R. = 5.52), and in the proximity of a sheep farm (O.R. = 2.72).

In contrast, the probability of capturing *P. perniciosus* showed a negative correlation with walls of bricks and mortar (O.R. = 0.26), non arable areas (O.R. = 0.27) and the proximity of pigeon and bee farms (O.R. = 0.15 and 0.1, respectively) (Table 2).

### Multivariate analysis

To construct the multivariate model, all the 24 variables with p <0.2 in the bivariate study were used. The variables that make up the multivariate logistic regression model and are shown to be the best predictors of the presence/absence of *P. perniciosus* are: an altitude of 51–150 m.a.s.l. (p = 0.01, O.R. = 8.6), location of the sampling sites at the edge of or between villages (p = 0.08, O.R. = 8.08), a south east orientation (p = 0.018, O.R. = 34.97), the absence of drainage holes with plastic pipes (p = 0.05, O.R. = 3.45), adjacent flora of garrigue shrubs (p = 0.001, O.R. = 38.05) and the proximity of a sheep farm (p = 0.001, O.R. = 20) (Table 3).

**Table 3:** **Risk factors for the presence of** ***Phlebotomus perniciosus*** **in Majorca: Multivariate logistic regression model**

	Odds ratio (I.C. 95 %)	p - Value
**Altitude (m.a.s.l.)**		**0.019**
0-50	**Ref.**	
51-150	8.653 (1.514 – 49.441)	0.015
>150	0.805 (0.131 – 4.964)	0.816
**Settlement**		
Within settlement	**Ref.**	
Edge of/between settlement	8.080 (1.737 – 37.596)	0.008
**Aspect**		**0.03**
Other (all orientations except south-east and west facing)	**Ref.**	
South-east-facing	34.975 (1.817 – 673.425)	0.018
West-facing	0.457 (0.116 – 1.798)	0.263
**Drainage hole construction**		
Plastic pipe	**Ref.**	
Other (unlined, cement pipe)	3.451 (1.002 – 11.880)	0.050
**Adjacent flora**		**0.001**
Aleppo pine and evergreen oaks	**Ref.**	
Garrigue shrubs	38.051 (4.900 – 295.469)	0.001
None	1.308 (0.323 – 5.307)	0.707
**Sheep farm near**		
No	**Ref.**	
Yes	19.989 (3.557 – 112.322)	0.001

Dependent variable presence/absence of *P. perniciosus*. Ref. Reference category. C. I. = Confidence interval. R^2^ = 0.571.

## Discussion

Four out of the five species previously reported for the island of Majorca (*P. perniciosus*, *P. ariasi*, *P. sergenti*, *P. papatasi* and *S. minuta*) [4, 12–14, 25] were captured. Although *P. ariasi* is cited [4, 13, 25], we were unable to capture this species despite sampling the whole island from 0 to 772 m.a.s.l and using a large number of traps. In Europe *P. ariasi* has been found at altitudes ranging from 10 m up to 2000 m.a.s.l. [20, 26], showing a preference for sub-humid or humid areas with cold winters (supra-Mediterranean) [21, 22, 27], while Majorca has a semi-arid and sub-humid climate with mild summers (meso- and thermo-Mediterranean). The repeated reporting of *P. ariasi* in Majorca may stem from an erroneous citing, which has been duplicated in other publications. Nevertheless, in this study, although captures were made throughout the whole island, they were restricted to the month of July (2008). Therefore, in order to assess more accurately whether *P. ariasi* is present or absent from the island, captures need to be made at different periods of sand fly activity. Also, intensive studies using CDC light traps should be carried out over 700 m a.s.l. in the mountainous regions of the island, particularly the area of the Serra de Tramuntana.

Among the species found, only *P. perniciosus* is a vector of *L. infantum*, and is responsible for human and canine leishmaniosis in the Mediterranean region [28, 29], while *P. sergenti* and *P. papatasi* are proven vectors of other *Leishmania* species in the Old World that are not present in Spain (*L. tropica* and *L. major*, respectively) [7, 30–32].

The most common sand fly species in Majorca is *S. minuta*, followed by *P. perniciosus*, *P. sergenti* and *P. papatasi.* The capturing method may have influenced the abundance level of each species, since it is known that sticky traps favor the capture of *S. minuta* females, which could be due to the feeding habits of this herpetophilic species and its preferred resting sites [24, 26]. Not enough *P. sergenti* and *P. papatasi* were captured for a statistical analysis of the factors affecting their presence in Majorca. As mentioned previously, most of the island is below 200 m.a.s.l., with a semi-arid climate, which are ideal conditions for *P. sergenti* to occur [33–35], yet this species was found at a low frequency (12.6 %). In other areas of Spain [35], *P. sergenti* has been found at altitudes of 0–1,153 m.a.s.l. and in the same type of meso- and thermo-Mediterranean bioclimates as in Majorca. Perhaps the location of traps within urbanized settlements (21 stations) or at the edge of/between settlements (90 stations), with little or no presence of humans, influenced the results, since *P. sergenti* is a peridomestic and anthropophilic species found in rural villages [30] and rare in intensely urban areas [36]. The other scarcely sampled species, *P. papatasi*, prefers peri-arid and Saharan environments [33], not present in Majorca.

*P. perniciosus* was captured in Majorca from 6 to 772 m a.s.l., the maximum altitude at which the sticky traps were placed, since above that there was a lack of appropriate locations for setting traps. In Europe, the species occupies sites from sea level to 1534 m a.s.l. [19, 20, 26]. The probability of finding *P. perniciosus* was significantly higher at altitudes of 51 – 150 m.a.s.l., both in the bivariate and multivariate analysis. Stations at 0 – 50 m.a.s.l. were located in breezy coastal areas and sand flies are very sensitive to windy conditions [26, 29, 30]. In locations at 51 – 150 m.a.s.l. the adjacent flora consisted principally of garrigue shrubs, where the probability of finding *P. perniciosus* is significantly higher.

Locations between or at the edge of settlements favored the presence of *P. perniciosus* compared to those within settlements, as found in other studies [1, 18, 19, 21], which would indicate that urban environments are not suitable for *P. perniciosus*. The barbicans and other locations where sticky traps were placed constituted resting sites, which are often near the larval breeding sites [22, 26, 29]. In agreement with the site location, a positive correlation was obtained with a rural agricultural and forestry environment, where the probability of finding *P. perniciosus* was 3 times higher than in a rural village, as well as with an area of agricultural land cover, where the probability was more than 5 times higher than in urbanized areas. These results also match the negative correlation found in non-arable points of capture, usually in rural and/or urbanized areas, where the probability of capturing *P. perniciosus* decreased in comparison with stations near arable areas (cereals). In non-urbanized areas the terrestrial cycle of immature forms would be favored, and the females would have more access to suitable oviposition sites [18, 21]. In addition, the deployment of insecticides in urbanized areas during the summer period when blood-sucking insects are active would reduce the population of sand flies in those settlements, and it is considered a way of controlling leishmaniosis [37].

The presence of animals near the sampling site increased the probability of encountering *P. perniciosus*, for several reasons: i) the presence of animal excrements would constitute a good sand fly breeding substrate; ii) sand flies have a poor capacity for flying and dispersing far from their breeding sites (usually 300 m and rarely more than1 km) [26, 29, 30], which may explain the existence of small localized populations [38]; and iii) *P. perniciosus* exhibits opportunistic feeding behavior [39–42]. Nevertheless, in contrast with previous studies [1, 18, 19], no correlation was found with the presence of animals or animal traces such as feces near the trapping sites, only with an abundance of animals in livestock farms. Not all livestock species attract *P. perniciosus* in the same way [19], and its capture increased significantly when sheep farms were near to the sampling site. Notably, sheep farms contain a greater number of animals that remain outside overnight, when sand flies are active. No demographic influence of humans or dogs was found, probably because the stations with the highest presence of *P. perniciosus* were located between villages, away from urban settlements.

Some other variables correlated with the presence of *P. perniciosus* only in the bivariate analysis, such as the type of road, site category, land cover, wall construction and arable area, while the type of drainage hole correlated only in the multivariate analysis. The probability of capturing *P. perniciosus* in a paved drive was 2.9 times higher than in a paved public road, where greater car traffic would disturb sand flies. Drainage holes in non-embankment walls favored the presence of *P. perniciosus* in contrast with those in embankments, probably because the former have no air currents. On the contrary, the presence of *P. perniciosus* decreased by 75% in stone or brick walls with mortar, probably because these have fewer suitable resting places than walls without mortar. As described elsewhere, the use of PVC in drainage holes decreased the probability of finding *P. perniciosus* and could be considered as a control method to reduce leishmaniosis transmission [19].

The influence of climate variables on the distribution and activity of sand flies has been repeatedly reported [26, 30, 31, 43]. In contrast with other reports [18, 19, 21, 41], in the current study in Majorca, climate variables did not affect the probability of finding *P. perniciosus*, probably due to the short period of time when captures were performed (July 2008) and the homogenous geographical conditions of most trapping sites. It should also be taken into account that the island of Majorca has a Mediterranean climate, which remains highly stable over long periods, with the exception of the mountainous areas, and captures were not made over 700 m.a.s.l., due to the absence of appropriate places to set traps. More studies involving periodic captures throughout the summer, or over one year are required, as has been done in another Balearic island (Minorca) [1], to obtain more data on the influence of climate conditions on sand fly distribution.

The presence of *P. perniciosus* in Majorca is a health issue since it is a vector of *L. infantum* in the Mediterranean area. Leishmaniosis poses a risk not only for the habitual inhabitants of the island, but also for the large numbers of tourists visiting in the summer, coinciding with the period of vector activity. In addition, these tourists often travel with their pets, which are at risk of developing CanL. In central and northern European countries cases of leishmaniosis have repeatedly been reported in humans and dogs that have visited endemic areas [43–45]. Recent accounts of sand flies with a proven or suspected capacity to transmit *L. infantum* in non-endemic areas [46, 47], together with the arrival of infected persons and animals, would favor the possibility of autochthonous transmission in new areas, as has been reported in the island of Minorca [1].

## Conclusion

The sand fly fauna in Majorca is composed of four species: *P. perniciosus*, P*. sergenti*, *P. papatasi* and *S. minuta*. The distribution of *P. perniciosus* extends throughout the island, from sea level to the mountains, being present in 70 % of the capture sites. This suggests that a risk of leishmaniosis transmission exists all over the island, and the presence of tourists during the period of *P. perniciosus* activity could favor the dispersion of the disease to other countries. The probability of finding *P. perniciosus* was higher at altitudes ranging from 51 to 150 m.a.s.l., with adjacent garrigue shrub vegetation, at the edge of or between settlements, and in proximity to a sheep farm.
